# Data quality of drug-resistant tuberculosis and antiretroviral therapy electronic registers in South Africa

**DOI:** 10.1186/s12889-019-7965-9

**Published:** 2019-12-05

**Authors:** Lise Jamieson, Denise Evans, Rebecca Berhanu, Nazir Ismail, Samantha Aucock, Kristina Wallengren, Lawrence Long

**Affiliations:** 1 Health Economics and Epidemiology Research Office (HE2RO), Department of Internal Medicine, School of Clinical Medicine, Faculty of Health Sciences, University of the Witwatersrand, Johannesburg, South Africa; 2 Department of Global Health, Boston University School of Public Health, Boston, USA; 3 Centre for Tuberculosis, National Institute of Communicable Diseases (NICD), Johannesburg, South Africa; 4 Department of Medical Microbiology, University of Pretoria, Pretoria, South Africa; 5 Tuberculosis & HIV Investigative Network (THINK), Doris Goodwin TB Hospital in Pietermaritzburg, Pietermaritzburg, South Africa

**Keywords:** EDRWeb, Tier,net, Data quality, Routine data

## Abstract

**Background:**

To assess the quality and completeness of treatment and outcome data in the electronic tuberculosis (TB) and antiretroviral treatment (ART) registers in drug-resistant (DR-) TB patients at three treatment facilities in South Africa.

**Methods:**

We did a retrospective cohort study using routinely-collected data from DR-TB registers of rifampicin resistant adults (≥18 years old), on ART, initiating DR-TB treatment between January 2012 and December 2013. We linked patient information from the DR-TB register to the ART register using patient identifiers and an algorithm based on string edit distance and date of birth. We describe data gaps and discrepancies found.

**Results:**

Overall, 2852 DR-TB patients met our inclusion criteria based on the DR-TB register data, and of these, 1685 (59%) could be matched to the ART registers. An additional 253 patients from the DR-TB registers were found in the ART registers, having initiated ART, despite the DR-TB register indicating that they were not on ART (or this data was missing). 11% of matched patients did not have TB treatment status recorded in the ART register despite being recorded as being on TB treatment in the DR-TB register, and 78% did not have an ART start date recorded in DR-TB register despite being on ART treatment as per the ART register. 11% of matched patients had a death recorded in one register but not the other, and of those with death recorded in both, 15% of dates differed by > 1 month.

**Conclusions:**

The underreporting of death and the lack of ART or TB status in the electronic DR-TB and ART registers could negatively impact monitoring efforts by downplaying the state of the TB/HIV epidemic. Improved recording of these data sources, and data integration across systems, could improve the accuracy of reporting for the national HIV/ART and TB programs.

## Background

South Africa has the largest population of patients on antiretroviral therapy (ART) at 4.36 million in 2017 [1]. Approximately 322,000 active tuberculosis (TB) cases and 16,000 new multi-drug resistant (MDR) or rifampicin-resistant (RR-) TB cases are estimated to occur each year [2]. The majority of the population affected by HIV and TB access their treatment through the public healthcare system. While health facilities keep their own paper records and registers, they are required to report into the national electronic registers for monitoring of HIV and TB. Two TB registers are widely in use: the national electronic TB register (ETR.Net) data for drug-sensitive TB and the electronic drug-resistant TB register (EDRWeb) for drug-resistant (DR)-TB. The Three Integrated Electronic Registers (TIER.NET) is a national monitoring/evaluation software system used to capture standardized data of patients on ART [3]. Introduced in 2011, it is used in over 3000 facilities in South Africa.

Effective monitoring of the national HIV and TB programmes relies on complete, accurate data. Knowing drug-resistant TB incidence within the HIV programme is critical. Furthermore, HIV infection rates and ART uptake amongst patients with TB are key indicators of TB and HIV programme integration. Accurate recording and reporting is crucial for programme monitoring, resource allocation, policy making and epidemiological analysis.

Though there was an evaluation of the implementation of TIER.NET [4] there have been no studies which assessed quality and completeness. A few studies have assessed the quality and completeness of the ETR. Net and EDRWeb [5–8]. A study of pediatric TB cases demonstrated that a third of these are never recorded in the ETR. Net [5], and another study found that in EDRWeb, 12% have missing HIV status and 4% have incorrect HIV status [8]. Although 90% of adult patients with TB are recorded in the ETR. Net [7], estimates for accurate reporting of HIV status range between 53.9 and 79% [6, 7] and reporting of ART status is as low as 24% [7]. In a study comparing ETR. Net to clinic source documents, agreement of ART status and TB treatment outcome was low at 59 and 47%, respectively [6]. A study comparing ETR. Net data to the National Health Laboratory Service data found 20% of smear positive TB cases were not registered in the ETR. Net and only 69.9% had patient records at the facilities from where the sample was sent [9].

We linked routinely-collected data from DR-TB and ART registers using patient identifiers (name, surname, date of birth, national identification number, if available). This paper aims to describe the methods used to match patients across routine data platforms, and data gaps and discrepancies found.

## Methods

### Data sources

In 2016/17 we extracted EDRWeb and TIER.NET data for two South African healthcare facilities. In addition to EDRWeb and TIER.NET, we included two additional sources of TB and HIV data, as these systems were used at a third healthcare facility we evaluated: the Focal Point Information System (FIS) is an electronic TB patient record system, allowing for multi-point data entry and is being used at three DR-TB treatment sites in South Africa. TherapyEdge-HIV™ (TE) is an electronic medical database system designed for HIV patient management currently used at six sites in South Africa. FIS and TE are more comprehensive than EDRWeb and TIER.NET in terms of variables collected. These systems were implemented at various time points at each site ranging from 2004 to 2013; at the time of data collection all the sites had a minimum of 3 years’ experience with their respective systems. In most instances data entry occurred from dedicated data staff at the site, but could also have been entered by other administrative or clinical staff. Some systems were donor funded and therfore had external support to implement and manage them (i.e. TE and FIS). TIER.NET and EDRWeb are government-mandated systems and implementation and management is through the South African National Department of Health (NDOH). To our knowledge there was no external validation or oversight of these electronic registers.

As this study could not have been done without knowing patient identifiers, a number of steps were taken to mitigate risks to patient confidentiality: 1) datasets were collected directly from each facility, password protected and transported on an encrypted hard-drive, 2) access to the data was limited to a single person who performed all analyses, 3) analytic datasets beyond the matching algorithm were de-identified for subsequent analyses.

### Study sites

The three facilities were based in the Gauteng and KwaZulu-Natal provinces. One is an outpatient Non-Governmental Organization (NGO)-supported DR-TB clinic at a public academic hospital. Another facility is a MDR-TB hospital providing inpatient and outpatient care. The third facility is an outpatient DR-TB centre. To maintain anonymity, we labelled them Site 1, 2 and 3. Site 1 uses the FIS and TE systems, while sites 2 and 3 use EDRWeb and TIER.NET.

Inclusion criteria for the study were: adults (aged 18+ years), laboratory diagnosis of rifampicin (RIF) resistant TB and initiated second-line TB treatment between January 2012–December 2013. HIV and ART status was primarily determined from either DR-TB or ART register. In both EDRWeb and FIS, ART status was recorded as a binary yes/no variable for ART started or as an ART start date, and either was used to identify ART status. Then, since the DR-TB registers may have incomplete HIV status and/or ART initiation information, we matched DR-TB patients with negative/missing HIV status, or HIV positive and not on ART (or whose ART status was unknown/missing) in the DR-TB register, to the ART register, in order to confirm HIV and/or ART status. Once HIV and ART status had been confirmed, subsequent analyses were restricted to patients who were HIV positive and on ART.

### Matching algorithm

We matched patients from the DR-TB register to their ART record by first merging those where the first letter of either the name or surname matched, and then using a generalized Levenshtein edit distance [10] to match names and surnames between the two datasets. The edit distance is a measure of similarity between two strings. Second or third names were also considered, if recorded. SAS version 9.3 was used for analysis, and the built-in function, COMPGED [11] was used.

Patients were marked as a match if their name in the one dataset matched their name in the other dataset with an edit distance of 100 or less [12, 13], and their birthdate matched. For patients with a missing date of birth, we matched on patient age and gender, alongside the names, allowing for a 1-year difference. For a birthdate match, we allowed for the year to shift up or down by 1 year, and we allowed for the month of birth to shift up or down by 1 month. We also allowed for the birthdate day and month to be erroneously switched in the datasets. Patients were considered a match if their South African identification number was an exact match. Then, in the case of an exact match of the surname and date of births between the two datasets, we were more lenient on the edit distance for the names (also for first, second or third name matches) by allowing for a score of 500 or less.

### Validation of matching

A random sample of 10% of each healthcare facility’s DR-TB patients meeting our inclusion criteria was selected. A manual matching process, considered the ‘gold standard’, was followed looking for patients in the corresponding ART register. We calculated sensitivity, specificity, positive predictive value (PPV) and negative predictive value (NPV).

### Statistical analysis

We compared demographics, TB characteristics and outcomes between those patients we could match and those we could not. We describe completeness and discrepancies of common variables in both ART and DR-TB registers. For reporting of death outcomes and ART start dates we calculated a Kappa statistic to measure agreement between the two data sources. The severity of date discrepancies was assessed by calculating the proportion of dates differing by > 1 month.

## Results

Overall, 2852 DR-TB adult patients, HIV+ on ART, initiated TB treatment between January 2012–December 2013 based on the DR-TB register data, and of these, 1685 (59%) could be matched to the ART registers. Of the 1526 patients who were reportedly not on ART (or the data was missing), 253 patients were found in the ART registers, having initiated ART. This brought our total matched up to 1938 (62%) and a total of 3105 DR-TB patients who were included in the subsequent analyses. While the rate of matching was high in Sites 1 and 2, with 76 and 80%, respectively, we could only match 59% of patients at Site 3. There were no substantial differences in age, gender, new treatment status, extra-pulmonary TB or TB outcome between those matched compared to those we couldn’t match to the ART register (Table 1), though the number of characteristics we could assess was limited.

**Table 1:** Differences between matched and unmatched cohort of DR-TB patients initiated on antiretroviral therapy

	All DR-TB/ART patients (*n* = 3105)	Not matched to ART data (*n* = 1167)	Matched to ART data (*n* = 1938)
Site, n (%)			
Site 1 (FIS/TE)	186 (6.0%)	37 (3.2%)	149 (7.7%)
Site 2 (EDRWeb/TIER.NET)	449 (14.5%)	109 (9.4%)	340 (17.5%)
Site 3 (EDRWeb/TIER.NET)	2470 (79.5%)	1021 (87.5%)	1449 (74.8%)
Gender, n (%)			
Female	1615 (52.1%)	614 (52.7%)	1001 (51.7%)
Male	1489 (48.0%)	553 (47.4%)	936 (48.3%)
Age, median (IQR)	35 (29–41)	35 (30–41)	34 (29–41)
Patient category			
New	1196 (38.5%)	430 (36.8%)	766 (39.5%)
Treatment after failure	1201 (38.7%)	465 (39.8%)	736 (38.0%)
Relapse	495 (15.9%)	198 (17.0%)	297 (15.3%)
Treatment after loss to follow-up	183 (5.9%)	64 (5.5%)	119 (6.1%)
Transfer-in	1 (< 0.1%)	1 (0.1%)	0 (0.0%)
Other	7 (0.2%)	4 (0.3%)	3 (0.2%)
Unknown	22 (0.7%)	5 (0.4%)	17 (0.9%)
TB type			
Extra-pulmonary TB	82 (2.6%)	21 (1.7%)	61 (3.1%)
Pulmonary TB	2672 (86.1%)	1045 (89.6%)	1627 (84.0%)
Missing	351 (11.3%)	101 (8.7%)	250 (12.9%)
TB outcome			
Cure	1038 (33.4%)	431 (36.9%)	607 (31.3%)
Completed treatment	661 (21.3%)	210 (18.0%)	451 (23.3%)
Death	557 (17.9%)	208 (17.8%)	349 (18.0%)
Treatment failure	72 (2.3%)	19 (1.6%)	53 (2.7%)
Lost to follow-up	689 (22.2%)	276 (23.7%)	413 (21.3%)
Transferred out	30 (1.0%)	13 (1.1%)	17 (0.9%)
Still on treatment	17 (0.5%)	2 (0.2%)	15 (0.8%)
Other	1 (0.0%)	0 (0.0%)	1 (0.1%)
Not specified	27 (0.9%)	3 (0.3%)	24 (1.2%)

*Abbreviations*: *DR-TB* Drug resistant tuberculosis, *ART* Antiretroviral therapy, *FIS* Focal Point Information System, *TE* TherapyEdge-HIV™, *EDRWeb* Electronic Drug Register Web, *TIER.NET* The Three Integrated Electronic Registers, *IQR* Interquartile range

In order to validate our matching algorithm, we randomly sampled 171 patients and compared the algorithm to our “gold standard” of manual matching. We found our algorithm to have a sensitivity of 87%, a specificity of 99%, a PPV of 98% and a NPV of 94%. The specificity was reduced by 1% and PPV by 2% due to an error in misclassification for one patient in the manual matching process.

Discrepancies between ART and DR-TB registers are presented in Table 2. Overall, 8.1% of patients had a discrepant date of birth, however the matching algorithm was more lenient on these if the name and surname were matched already; another 2.5% of patients had a date of birth missing in either register so these patients would have been matched on age, alongside gender and names. All patients in Site 1 had their HIV status recorded as HIV positive in the DR-TB register, but 6.2 and 4.5% of patients in Sites 2 and 3, respectively, had either a negative, unknown or missing HIV status in the DR-TB register, despite being initiated on ART. Overall, 8.7% of patients had no record of TB treatment (e.g. missing) in the ART register data and 2.5% of patients were recorded as not being on TB treatment (e.g. TB treatment incorrectly recorded as “No”) in the ART register record. Of the 217 patients whose TB treatment status was incorrectly recorded in the ART register, 82% had initiated ART prior to TB treatment and thus this is likely due to the records not being updated. However, of all patients who had initiated ART prior to TB treatment (61%, *n* = 1187), majority (85%) had TB treatment status recorded correctly, demonstrating that most records had been updated in real time.

**Table 2:** Discrepancies in data found between ART and DR-TB registers in patients matched between data sources

	Site 1 (FIS/TE) (*n* = 149)	Site 2 (EDRWeb/ TIER.NET) (*n* = 340)	Site 3 (EDRWeb/ TIER.NET) (*n* = 1449)	Total (*n* = 1938)
Reporting of date of birth				
Agree	92.6% (138)	92.7% (315)	88.3% (1279)	89.4% (1732)
Discrepant between DR-TB and ART register	7.4% (11)	7.4% (25)	8.4% (121)	8.1% (157)
Missing in ART register	0.0% (0)	0.0% (0)	0.1% (2)	0.1% (2)
Missing in DR-TB register	0.0% (0)	0.0% (0)	3.2% (47)	2.4% (47)
DR-TB treatment recorded in ART register				
Not recorded/missing	3.4% (5)	42.4% (144)	1.4% (20)	8.7% (169)
Has not started TB treatment	0.0% (0)	2.7% (9)	2.7% (39)	2.5% (48)
Started TB treatment	96.6% (144)	55.0% (187)	95.9% (1390)	88.8% (1721)
HIV status reported in DR-TB register				
Positive	100% (149)	93.8% (319)	95.6% (1385)	95.6% (1853)
Negative	0.0% (0)	5.6% (19)	2.6% (37)	2.9% (56)
Unknown or missing	0.0% (0)	0.6% (2)	1.9% (27)	1.5% (29)
ART status recorded in DR-TB register				
Not recorded/missing	0.0% (0)	1.5% (5)	3.7% (53)	3.0% (58)
Has not started ART	6.7% (10)	5.6% (19)	11.7% (169)	10.2% (198)
Started ART	93.3% (139)	92.9% (316)	84.7% (1227)	86.8% (1682)
Reporting of ART start date				
Not recorded in either register	1.3% (2)	0.0% (0)	0.1% (2)	0.2% (4)
Recorded in ART register, but not DR-TB register	6.7% (10)	99.7% (339)	80.5% (1167)	78.2% (1516)
Recorded in DR-TB register, but not ART register	10.7% (16)	0.0% (0)	0.0% (0)	0.1% (16)
Recorded in both registers	81.2% (121)	0.3% (1)	19.3% (280)	20.7% (402)
ART start dates agree	32.2% (39/121)	0.0% (0/1)	84.6% (237/280)	68.7% (276/402)
ART start dates differ by ≤1 month	17.3% (21/121)	0.0% (0/1)	5.4% (15/280)	9.0% (36/402)
ART start dates differ by > 1 month	50.4% (61/121)	100% (1/1)	10.0% (28/280)	22.4% (90/402)
Reporting of ART regimen				
Not recorded in either register	8.1% (12)	0.0% (0)	0.0% (0)	0.6% (12)
Recorded in ART register, but not DR-TB register	44.3% (66)	100% (340)	100% (1449)	95.7% (1855)
Recorded in DR-TB register, but not ART register	3.4% (5)	0.0% (0)	0.0% (0)	0.3% (5)
Recorded in both registers	44.3% (66)	0.0% (0)	0.0% (0)	3.4% (66)
ART regimens agree	86.4% (57/66)	N/A	N/A	86.4% (57/66)
Reporting of death and date of death				
No deaths in either register	81.9% (122)	84.4% (287)	76.8% (1113)	78.5% (1522)
Death in ART register, but not DR-TB register	2.7% (4)	1.5% (5)	4.0% (58)	3.5% (67)
*Reported in DR-TB register as:*				
*Lost to follow-up*	3	5	30	38
*Transferred out*	1	0	0	1
*Cured*	0	0	8	8
*Treatment completed*	0	0	6	6
*Still on treatment*	0	0	2	2
*Treatment failure*	0	0	1	1
*Not specified*	0	0	11	11
Death in DR-TB register, but not ART register	4.7% (7)	2.9% (10)	8.6% (124)	7.3% (141)
*Reported in ART register as:*				
*Lost to follow-up*	0	8	106	114
*Transferred out*	3	2	16	21
*Not specified*	4	0	0	4
Death in both registers	10.7% (16)	11.2% (38)	10.6% (154)	10.7% (208)
Death dates agree	25.0% (4/16)	55.3% (21/38)	59.1% (91/154)	55.8% (116/208)
Death dates differ by ≤1 month	31.3% (5/16)	39.5% (15/38)	26.0% (40/154)	28.8% (60/208)
Death dates differ by > 1 month	43.8% (7/16)	5.3% (2/38)	14.9% (23/154)	15.4% (32/208)
Death reported in ART register prior to non-death outcome in DR-TB register	0.0% (0)	0.3% (1)	1.5% (22)	1.2% (23)
Death reported in DR-TB register prior to non-death outcome in ART register	0.0% (0)	0.9% (3)	3.4% (49)	2.7% (52)
Lost to follow-up in ART register, death recorded in DR-TB register within 3 months	0.0% (0)	1.8% (6)	4.6% (66)	3.7% (72)
Lost to follow-up in DR-TB register, death recorded in ART register within 3 months	2.0% (3)	0.9% (3)	0.5% (7)	0.7% (13)
Transferred out in ART register, death recorded in DR-TB register within 3 months	0.7% (1)	0.3% (1)	0.4% (5)	0.4% (7)
Transferred out in DR-TB register, death recorded in ART register within 3 months	0.0% (0)	0.0% (0)	0.0% (0)	0.0% (0)

*Abbreviations*: *DR-TB* Drug resistant tuberculosis, *ART* Antiretroviral therapy, *FIS* Focal Point Information System, *TE* TherapyEdge-HIV™, *EDRWeb* Electronic Drug Register Web, *TIER.NET* The Three Integrated Electronic Registers

Majority (87%) had ART status correctly recorded in the DR-TB register. While ART start date was reported in both ART and DR-TB registers for most patients at Site 1 (81.2%), 67.8% were discrepant, and three quarters of those discrepant differed by > 1 month. At Site 1, 12% of patients had a missing ART start date in the ART register, though the data indicated they initiated treatment. Most patients at Sites 2 and 3 did not have ART start date recorded in the DR-TB register (99.7 and 80.5%, respectively). Of the 280 patients at Site 3 that did have ART start date recorded in both registers, 15.4% had discrepant dates with 68.7% of ART start dates differing by > 1 month. Overall there was poor agreement of the reporting of the ART start date between the ART and DR-TB registers (Kappa = 0.001). Sites 2 and 3 did not have ART regimen information recorded in the DR-TB register so we could not compare between the two datasets. At Site 1, where ART regimen was recorded in both DR-TB and ART registers for 44.3% of patients, it was in agreement for majority of them (86.4%). However, 11.4% of patients at Site 1 did not have ART regimen recorded in the ART register.

Overall, 7.3% of patients had a death reported in the DR-TB register but not the ART register. Of these 80.9% were reported in the ART register as a lost to follow-up, 14.9% as transferred out, and 2.8% of patients had no outcome reported. While these may be explained by the timing of when the information gets recorded, a large proportion of these deaths (45.4%) were reported as deaths in the DR-TB register less than 3 months after the non-death outcome in the ART register and 36.9% had dates which occurred prior to the non-death outcome date in the ART register.

Overall, 3.5% had a death reported in the ART register but not the DR-TB register. Of these, 56.7% were reported as lost to follow-up in the DR-TB register, 16.4% had no outcome reported, and one patient was reportedly transferred out. The remaining patients had TB treatment-specific outcomes reported: 11.9% cured, 9.0% completed treatment, 3.0% still on treatment and one patient had failed TB treatment. Of the 67 patients with death reported in the ART register and not the DR-TB register, 29.9% were reported as deaths in the ART register < 3 months after the non-death outcome in the DR-TB register and 34.3% had their death dates prior to the non-death outcome date in the DR-TB register. Overall agreement between reporting of death was good between the DR-TB and ART registers (Kappa = 0.60).

## Discussion

The electronic DR-TB and ART registers, used by majority of public healthcare facilities across South Africa, are crucial for monitoring treatment programme outcomes and have improved the quality of the reporting to the South African NDOH and international organizations such as the World Health Organization. They have also allowed for data access by various research organizations. However, they do not come without their weaknesses. Our analysis has shown an underreporting of crucial indices of patient outcomes, in particular ART start dates and death outcomes. Close to 11% of patients had a death reported in the DR-TB register but not the ART register, or vice versa. Majority of the corresponding non-death outcomes were reported as lost to follow-up, thus we can assume the patient was marked as such when they failed to return to the facility. Though these are likely explained by the design or timing of data entry in either system, it still remains problematic to researchers and government who analyze these individual data systems at face value for monitoring purposes; additional follow-up is required to ensure that death does not go underreported. A possible solution to confirming death outcomes would be to have the electronic registers linked to the South African national death registry, however this will depend on having a valid South African identity number correctly recorded, and with the large patient numbers involved, it would need to be an automated and continuous procedure. We also found poor quality of recording of ART information in EDRWeb. The majority of patients at the two study sites that used EDRWeb exclusively did not have ART start date recorded (99.7 and 80.5%, respectively). Though FIS also had missing ART information, it was on a smaller scale compared to EDRWeb. ART start dates and ART regimen are important to know for monitoring of the DR-TB programme, as well as monitoring drug interactions as new drugs are being introduced, for example dolutegravir for ART and bedaquiline for TB. Rates of DR-TB diagnoses in the ART programme are also underreported (as with Site 2 where just under half of patients did not have DR-TB status recorded in the ART register). Importantly, these discrepancies existed across sites and different systems, which may suggest that this could be a problem for other similar healthcare facilities across South Africa.

Our matching algorithm performed reasonably well in linking patients between datasets, however it is limited by the quality of the reporting of patient identifiers, and the sensitivity of the algorithm could be improved if the patient identifiers were accurately recorded in both registers. This highlights the need for a unique patient identifier within the public health system, something planned for in the National Health Insurance Policy proposed by the South African government [14].

## Conclusions

The quality and completeness of the electronic DR-TB and ART registers is concerning for researchers and government who use it to monitor the outcomes of the South African TB and HIV programmes. Although South Africa’s public healthcare system is understaffed and overburdened and regular internal auditing of data may not be feasible, urgent attention is needed to address data gaps in the ART and DR-TB electronic systems.

In 2016, the NDOH implemented a TB module into TIER.NET aiming to integrate the TB and HIV data to increase reporting efficiency. While this TB module can capture DR-TB through drug sensitivity and TB regimen variables, EDRWeb will still be used to monitor DR-TB patients. The impact of the TIER.NET TB module is yet to be determined, and while data integration can improve the quality of these data systems, accurate reporting is still crucial to ensure good data quality and their ability to have a meaningful impact on monitoring, policy, planning and epidemiological analysis. Furthermore, it is essential to have intensive, ongoing training of staff who capture data into electronic registers, constant emphasis on data quality, improvement of patient file management, and the transfer of information to the data capturing team.

## Data Availability

The data is owned by the study site and National Department of Health (South Africa) and governed by the Human Research Ethics Committee (University of Witwatersrand, Johannesburg, South Africa). All relevant data is included in the paper and supplementary tables. The full data are available from the Health Economics and Epidemiology Research Office for researchers who meet the criteria for access to confidential data and have approval from the owners of the data (information@heroza.org).

## Abbreviations

ART: Antiretroviral therapy
DR-TB: Drug resistant tuberculosis
EDRWeb: Electronic Drug Register Web
ETR.Net: Electronic TB register
FIS: Focal Point Information System
HIV: Human immunodeficiency virus
IQR: Interquartile range
MDR: Multi-drug resistant
NDOH: National Department of Health
NGO: Non-Governmental Organization
NPV: Negative predictive value
PPV: Positive predictive value
RR: Rifampicin-resistant
TB: Tuberculosis
TE: TherapyEdge-HIV™
TIER.NET: The Three Integrated Electronic Registers
